# fNIRS Assessment of Speech Comprehension in Children with Normal Hearing and Children with Hearing Aids in Virtual Acoustic Environments: Pilot Data and Practical Recommendations

**DOI:** 10.3390/children7110219

**Published:** 2020-11-07

**Authors:** Laura Bell, Z. Ellen Peng, Florian Pausch, Vanessa Reindl, Christiane Neuschaefer-Rube, Janina Fels, Kerstin Konrad

**Affiliations:** 1 Child Neuropsychology Section, Department of Child and Adolescent Psychiatry, Psychosomatics and Psychotherapy, Medical Faculty, RWTH Aachen University, 52074 Aachen, Germany; vreindl@ukaachen.de (V.R.); kkonrad@ukaachen.de (K.K.); 2 Teaching and Research Area of Medical Acoustics, Institute of Technical Acoustics, RWTH Aachen University, 52074 Aachen, Germany; florian.pausch@akustik.rwth-aachen.de (F.P.); Janina.Fels@akustik.rwth-aachen.de (J.F.); 3 Waisman Center, University of Wisconsin-Madison, Madison, WI 53705, USA; z.ellen.peng@wisc.edu; 4 JARA-Brain Institute II, Molecular Neuroscience and Neuroimaging, RWTH Aachen & Research Centre Juelich, 52428 Juelich, Germany; 5 Clinic of Phoniatrics, Pedaudiology, and Communication Disorders, Medical Faculty, RWTH Aachen University, 52074 Aachen, Germany; cneuschaefer@ukaachen.de

**Keywords:** functional near-infrared spectroscopy, virtual acoustics, speech perception, multimethod approach, virtual acoustic environments

## Abstract

The integration of virtual acoustic environments (VAEs) with functional near-infrared spectroscopy (fNIRS) offers novel avenues to investigate behavioral and neural processes of speech-in-noise (SIN) comprehension in complex auditory scenes. Particularly in children with hearing aids (HAs), the combined application might offer new insights into the neural mechanism of SIN perception in simulated real-life acoustic scenarios. Here, we present first pilot data from six children with normal hearing (NH) and three children with bilateral HAs to explore the potential applicability of this novel approach. Children with NH received a speech recognition benefit from low room reverberation and target-distractors’ spatial separation, particularly when the pitch of the target and the distractors was similar. On the neural level, the left inferior frontal gyrus appeared to support SIN comprehension during effortful listening. Children with HAs showed decreased SIN perception across conditions. The VAE-fNIRS approach is critically compared to traditional SIN assessments. Although the current study shows that feasibility still needs to be improved, the combined application potentially offers a promising tool to investigate novel research questions in simulated real-life listening. Future modified VAE-fNIRS applications are warranted to replicate the current findings and to validate its application in research and clinical settings.

## 1. Introduction

### 1.1. The Influence of Hearing Loss and Auditory Noise on Development

Hearing plays a crucial role in children’s development when learning through verbal communication. Yet, critical learning is often challenged by noise [1,2,3,4]. Hearing loss (HL) presents additional challenges, particularly for infants and young children. Even with early interventions such as hearing aids (HAs), children with HL are at higher risk of delays in language development than children with normal hearing (NH; [5,6,7,8]). The current clinical fitting of HAs focuses primarily on ensuring audibility in quiet environments [9,10]. It does not, however, directly address how children listen in complex acoustic environments, such as classrooms. Indoor environments introduce reverberation that distorts auditory cues by excessive sound reflections [11,12]. Reverberation and multi-talker background noise have been shown to negatively affect speech-in-noise (SIN) comprehension in children with NH and children with HL in the classroom [4,13]. Yet, while auditory spatial cues enable speech stream segregation in children with NH [14,15], the lack of reliable access to spatial hearing through hearing devices presents the biggest challenge to date for children fitted with bilateral HAs (i.e., one device in each ear). Access to binaural information, i.e., interaural time and intensity differences, which enable binaural release from masking for children with NH, can be distorted or absent in individuals with HL [16]. Even with aided hearing through cochlear implants (CIs) or HAs, speech recognition in noise rarely reaches the levels of children with NH. For example, it has been shown that children with HAs achieved a spatial release from masking similar to those of children with NH when speech and background noise emanated from a frontal source, but performed poorly when a spatial separation from background noise and target was introduced [17]. Children with HL often require a better signal-to-noise ratio (SNR) to achieve the same level of performance. Particularly for children with HAs, cognitive and language abilities appear to strongly influence their level of SIN comprehension [17,18].

### 1.2. The Ease of Language Understanding Model

According to the Ease of Language Understanding (ELU) model, speech recognition depends on a complex interaction of language signals, working memory capacity, executive functions, and episodic as well as long-term memory [19,20]. In ideal, i.e., quiet and simple, listening conditions, auditory signals match phonological representations in the mental lexicon and words can thus be easily and implicitly comprehended. In complex, noisy environments or in the case of HL, however, explicit linguistic and working memory abilities are demanded [19]. While this model has been shown to hold for adults (e.g., for a review, see Rönnberg, Holmer, and Rudner [20]), it is not fully understood how mental representations are developed and how the behavioral outcomes of SIN perception are affected by HL, HAs, and auditory noise among young children that are still undergoing language development and have lower linguistic and working memory abilities. Based inter alia on findings in the field of sign language, the developmental ELU model (D-ELU) has been proposed. The D-ELU model suggests that domain general and language-specific skills are required during working memory processing to establish new and revised cognitive representations in children [21,22]. While children with NH might thus already be able to devote working memory capacity for the establishment and storing of novel representations based on semantics in complex, noisy environments, children with HL might have to use most of their working memory capacity to predict words based on phonology and existing lexical abilities of the novel but also already known representations (e.g., as indicated by a study by Signoret and Rudner [23]). This might also explain why children with HL and stronger vocabulary and working memory abilities often perform better than their peers with poorer skills and HL in noisy situations [24]. Further investigations that mimic naturalistic environments are warranted to advance our understanding of these mechanisms and to identify the underlying neural processes that support speech in noise recognition, particularly in children with HL and residual hearing capacity.

### 1.3. Behavioral Speech-In-Noise Comprehension Assessments

In the past, a variety of behavioral tests have been designed to assess SIN recognition in adults and some for application in children. Examples of such tests are the Hearing In Noise Test (HINT; Nilsson et al. [25]), the Words-in-Noise test (WIN; Wilson [26]), and the Listening in Spatialized Noise-Sentences test (LISN-S; Cameron and Dillon [27]). See Table 1, category A for further examples of speech recognition tests.

For listeners, many factors are known to affect SIN comprehension and can be varied within these assessments. These might include the speech stimuli and the distractor, such as competing talkers. For example, in the LISN-S for children, the location of the maskers (0° vs. 90° azimuth) as well as the pitch similarity to the target speaker (same as or different from the target speaker; [27]) are varied. While several of the SIN tests have been applied to typically developing children and translated into several languages [70,71], a recent study showed that language skills were a significant predictor for performance in the Hearing In Noise Test (HINT) for children with CI, HAs, and a developmental language disorder, but not children with NH [71]. To investigate whether early executively demanding (linguistic) training might help children with HL to compensate and to better understand the mechanisms supporting SIN comprehension, it is important to create appropriate testing environments that mimic complex real-world auditory situations and are appropriate for the assessment of children with HL. Such testing environments might guide the future design of audiological assessment tools that are applicable to daily listening in quiet and in noisy environments.

### 1.4. Speech Comprehension and Virtual Acoustic Reality

Recent advances in acoustic virtual reality enable reliable application of increasingly plausible virtual auditory environments (VAEs) for laboratory-based hearing research [42,72,73,74,75]. By manipulating auditory cues in VAEs, various factors influencing speech comprehension can be examined in isolation. Past studies have used VAEs to explore spatial hearing in free-field environments, such as sound localization [33,34], auditory distance perception [40,41,42,43], and auditory attention switching [45,46]; see Table 1, category B. In addition to free-field listening, recent VAE studies also began to examine more realistic indoor auditory environments, such as speech understanding in noisy classrooms ([47,48,49]; Table 1, category B). Thereby, SIN tests can be assessed in simulated real-life settings. While most current VAE work mainly focused on individuals with NH, there is an increasing interest in VAE application to evaluate outcomes of assistive hearing devices [24,50,53,76].

### 1.5. Speech Comprehension and Functional Near-Infrared Spectroscopy

Next to assessments that mimic real-life listening scenarios, it is of interest to gain insights into the underlying neural processes that contribute to good SIN perception and individual differences in speech comprehension. Table 1, category C provides examples of past neuroimaging studies on word and speech (in noise) understanding utilizing different neuroimaging techniques. Functional near-infrared spectroscopy (fNIRS) has recently gained much traction as a versatile optical neuroimaging tool to assess auditory paradigms and language development in both NH listeners, and those fitted with Cis [57,77,78,79,80,81,82,83]. A current study also looked at auditory mechanisms in children fitted with HAs [84]. fNIRS is particularly suitable for investigations of auditory paradigms due to its silent operation, higher spatial resolution than electroencephalography (EEG), fewer motion restrictions, and compatibility with hearing device use in contrast to functional magnetic resonance imaging [85]. Neural activity is inferred by the continuously recorded changes in oxygenated, deoxygenated, and total hemoglobin concentration (∆HbO, ∆HbR, ∆HbT). Essentially, fNIRS allows the capturing of the relation between speech recognition and cortical activation. In previous studies, superior temporal gyrus (STG) activity was considered predictive of speech comprehension [54,86,87]. Next to the temporal cortices, the left inferior frontal gyrus (IFG) has been shown to facilitate the differentiation of an auditory stream of interest from auditory noise during effortful listening, which requires a higher cognitive load [54,55,56,57,58,88]. With a variety of auditory tasks applied in the previous literature, fNIRS has thus demonstrated its potential for combination with VAEs to elucidate the underlying neural mechanisms of speech comprehension during real-world listening.

### 1.6. A Novel Approach to Elucidate SIN Comprehension: A VAE-fNIRS Application

Table 1 provides examples of past tests and studies that investigated auditory processing and SIN comprehension by means of behavioral-only, VAE-based, or neuroimaging assessments. In the current pilot study, we introduce a novel experimental approach to investigate how children with NH and children with HAs utilize auditory cues to understand SIN in complex simulated auditory environments. While children are exposed to a virtual acoustic simulation of a realistic classroom environment from VAE, our rigorous approach combines a simultaneous behavioral assessment of SIN performance and a neural measure through fNIRS. After a detailed description of the methods and testing equipment, its first application is illustrated by pilot data. Clear recommendations are provided to address current challenges of the novel approach.

## 2. Materials and Methods

### 2.1. Participants

**Figure 1:**
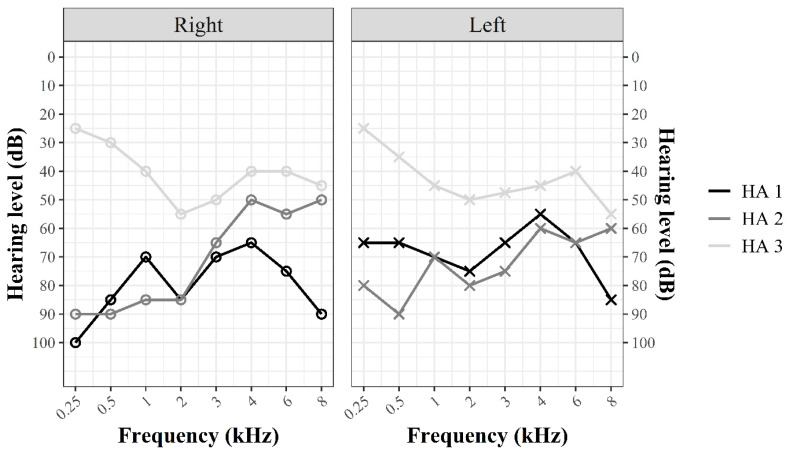
Air-conduction pure tone audiograms. The air-conduction pure tone audiograms for (**left**) and (**right**) ear of each child in the hearing aid (HA) group are depicted.

Data of six children with NH (*M* = 10.15, *SD* = 1.43; 6 male), and three children (2 female) fitted with bilateral HAs (*M* = 10.37, *SD* = 1.68), who completed all test conditions with good quality fNIRS measurements (see Section 2.4.2 for the details on the data quality check), are reported in this pilot study. HAs were worn on average 12.50 h (*SD* = 0.41) on a school day and 12.66 h (*SD* = 1.70) on a day out of school. Figure 1 depicts the individual unaided pure tone audiograms that were obtained within three weeks of the study participation for the HA group. For all NH children, NH was based on the mandatory early hearing screen (U9, including headphone-based audiometry) and parental report on the day of testing. Before participation, parents provided written informed consent and children’s assent. The study was approved by the local ethical committee (Medical Faculty, University Hospital Aachen; EK 188/15) and conducted in accordance with the Code of Ethics of the World Medical Association (Declaration of Helsinki). See Supplementary Table S2 for information on the demographic and hearing assessment.

### 2.2. Equipment and Virtual Acoustic Environment

**Figure 2:**
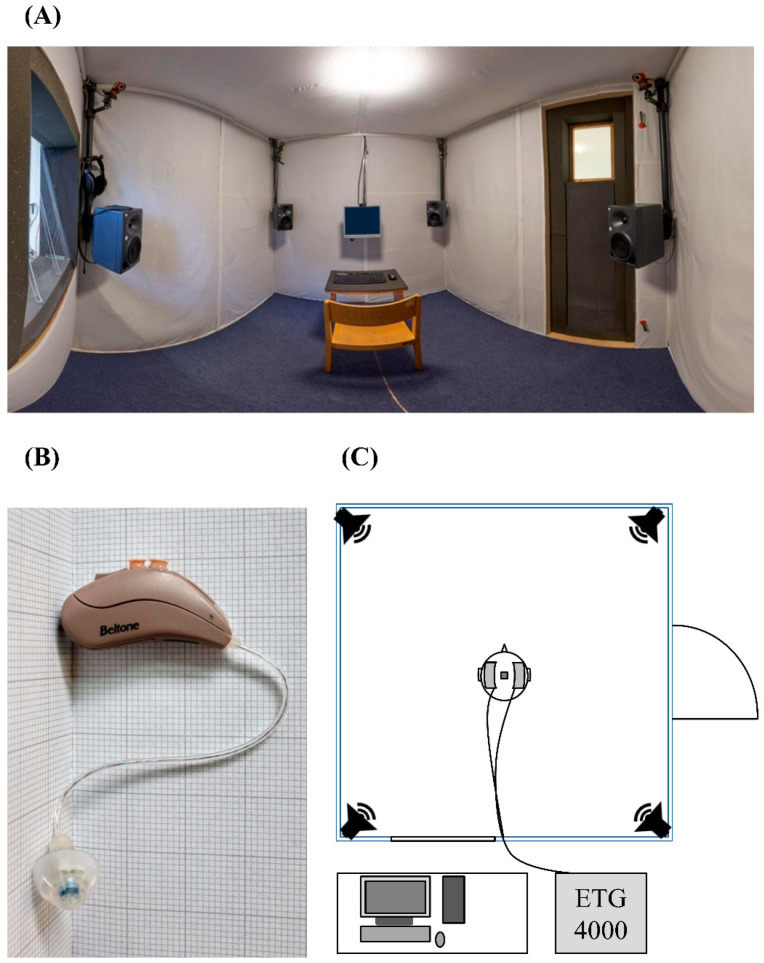
Illustration of the experimental setting. Panorama view of the inside of the sound insulated booth (**A**), left behind-the-ear receiver-in-canal device used as research hearing aids (HAs) (**B**), and schematic illustration of the setup (**C**). The participant was seated centrally within the sound insulated booth. For a subset of participants in the HA group, research HAs were led into the sound insulated booth. The fNIRS fibers and optodes were directed into the booth to be placed on the participant’s head within a cap. On top of the cap, the rigid body base, holding reflective markers, was mounted. The cameras, tracking the motion, were positioned in each corner above the loudspeakers. The computer and ETG4000 (Hitachi Medical Corporation, Tokyo, Japan) were placed outside the booth to minimize equipment noise.

See Supplementary Table S1 for a detailed overview of all testing equipment. Testing was performed in a custom-built sound attenuated booth (L × W × H = 2.12 × 2.12 × 2 m; 9 m^3^; Figure 2A,C). A four-channel loudspeaker array (Neumann KH-120A; Georg Neumann GmbH, Berlin, Germany) was positioned at ear height, one at each corner, for audio playback using crosstalk cancellation [89]. The child was seated in the center of the booth with a distance of 110 cm from each loudspeaker.

The behavioral paradigm was implemented in a simulated virtual classroom (L × W × H = 11.8 × 7.6 × 3 m, V = 244 m^3^). All room acoustic simulations were performed in the real-time auralization framework Room Acoustics for Virtual Acoustic Environments (RAVEN; Pelzer, Aspöck, Schröder and Vorländer [75], Pausch et al. [90], Schröder [91]). To achieve a realistic spatial percept, VAEs can be rendered using head-related transfer functions (HRTFs). Accounting for differences in head size between children and adults and enhanced physical correctness of the spatial cues delivered in VAEs [92,93], an individualization procedure was applied for each child. The individualization procedure was achieved by scaling the HRTFs [94,95] from an adult artificial head [96], which were measured at a spatial resolution of 1° × 3° in azimuth and elevation angles. Merged with the room acoustic simulations, the scaled HRTFs were used to create the binaural stimuli. The software Virtual Acoustics was utilized for the binaural real-time reproduction. The acoustic simulation was updated for the current position and orientation of the child’s interaural axis center based on the input of an optical motion tracking system (Flex 13, OptiTrack, Corvallis, OR, USA).

In addition to acoustic transmission through loudspeakers, the system also included a pair of research HAs (custom-made behind-the-ear receiver-in-canal devices with open fitting by GN ReSound, Ballerup, Denmark; Figure 2B) to play the auditory stimuli for children using HAs. This combined reproduction strategy aims at approaching the real-life equivalent, where individuals are likely to use their residual hearing. The simulated HA microphone signals were based on scaled hearing aid-related transfer functions (HARTFs; Pausch, Aspock, Vorlander, and Fels [90]). Together with the results of the room acoustic simulation, they contained all spatial signal characteristics as they would be captured by the front HA microphones in the virtual classroom. To address the real-life delay in HA signal processing, a variable delay line added a 5 ms delay relative to the binaural loudspeaker reproduction at the ear drum level [97]. Using the simulated signals as input, a MATLAB-based [98] real-time software platform for the emulation of HA algorithms with individual fitting capability was integrated [99].

For this pilot study, the software platform was utilized for one child in the HA group, using gain prescription based on the individual’s unaided audiogram [100]. No directional HA algorithms or other signal enhancement algorithms were included. The other two children were unable to use the research HAs due to their higher degree of HL that would have required amplification that exceeded the safety limits in the software platform. Instead, they were listening with their own HAs to the VAEs reproduced via the loudspeakers. Assuming negligible residual hearing capabilities for these individuals, the binaural playback over loudspeakers with CTC filters was based solely on individually scaled HARTFs instead of a mixture of playback HRTFs (loudspeaker playback) and HARTFs (HA playback) as in combined reproduction. All children with NH received binaural stimulus playback only via loudspeakers with CTC filters based on individually scaled HRTFs.

**Figure 3:**
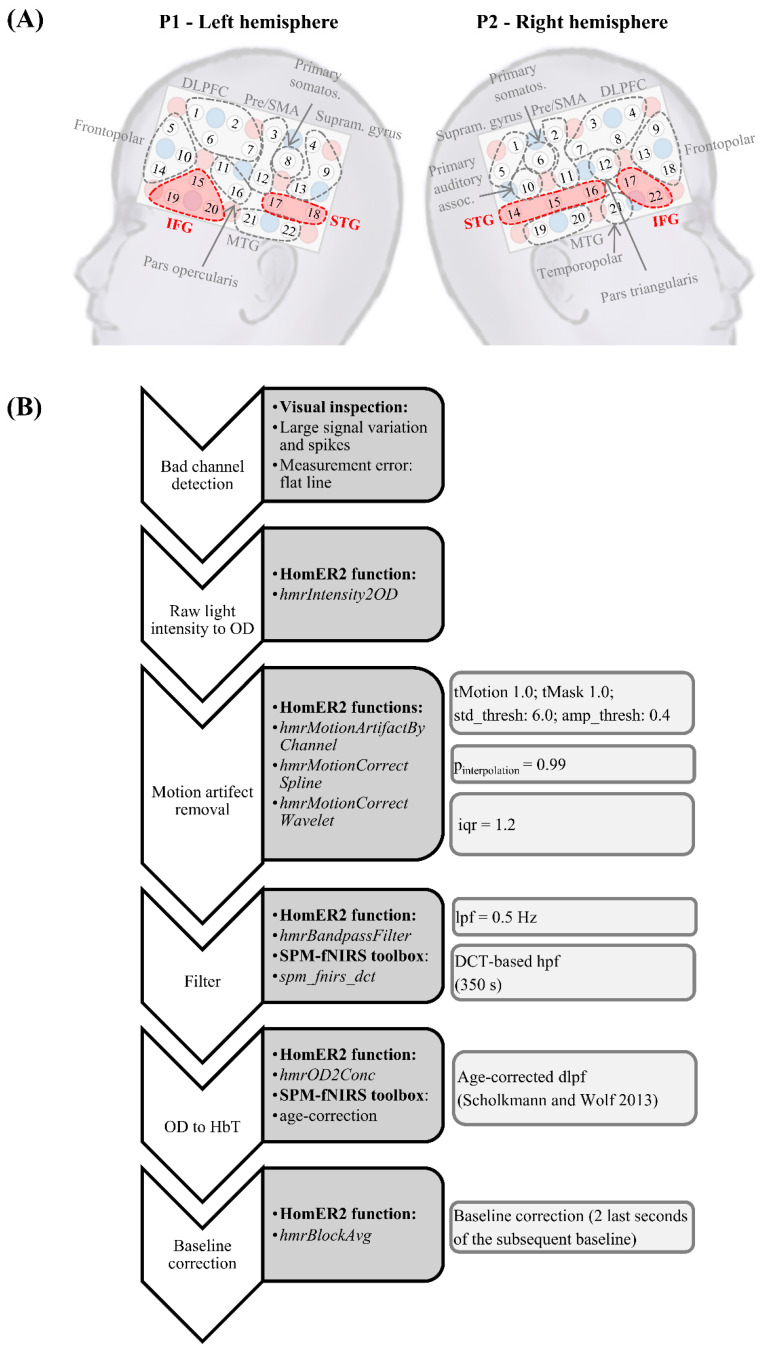
The fNIRS setup. (**A**) The fNIRS configuration is illustrated on a head model and the underlying brain areas are marked as areas surrounded by dashed lines. The round, unnumbered dots represent sending and receiving fNIRS optodes. Numbered circles in between unnumbered dots represent a measurement channel. (**B**) fNIRS preprocessing pipeline. Abbreviations: P—Probe set; LH—left hemisphere; RH—right hemisphere; DLPFC—dorsolateral prefrontal cortex; IFG—inferior frontal gyrus; pre/SMA—premotor and supplementary motor area; STG—superior temporal gyrus; MTG—medial temporal gyrus; primary somatos.—primary somatosensory area; primary auditory assoc.—primary auditory association area; supram. gyrus—supramarginal gyrus; CoV—coefficient of variation; OD—optical density; lpf—low pass filter; DCT—discrete cosine transformation; hpf—high pass filter; dlpf—differential path length factor.

To minimize equipment noise, the computer and fNIRS system (ETG4000, Hitachi Medical Corporation, Tokyo, Japan) were placed outside the booth. To ensure firm hold, the 2 × 3 × 5 fNIRS probe holders with 2 × 22 measurement channels (CHs) were placed in an EEG cap (Easycap GmbH, Herrsching, Germany). The probe sets were positioned symmetrically on the left and right side of the head (Figure 3A). The last receiving optode of the lowest row was placed above the ear (proximal to T3/T4 of the 10/20 system [101]). The anterior, lower corner of each probe set was directed towards the end of the eyebrows. A virtual registration approach was applied [102], with optode positions resembling 2 × 3 × 5 CH configurations by the Jichi Medical University [103]. The 2 regions of interest (ROIs), STG and IFG, are based on anatomical labels of the highest probability (Figure 3A). Changes in HbO, HbR, and HbT were obtained via 2 wavelengths (695 and 830 nm) and a sampling frequency of 10 Hz. HbT has been suggested to be less susceptible to pial vein contamination [104] and 2 large pial veins (the superior anastomotic and superficial middle cerebral vein) underlie our fNIRS configuration. Therefore, the current exploratory analyses of the pilot data focused on ∆HbT.

### 2.3. Experimental Design and Procedure

**Figure 4:**
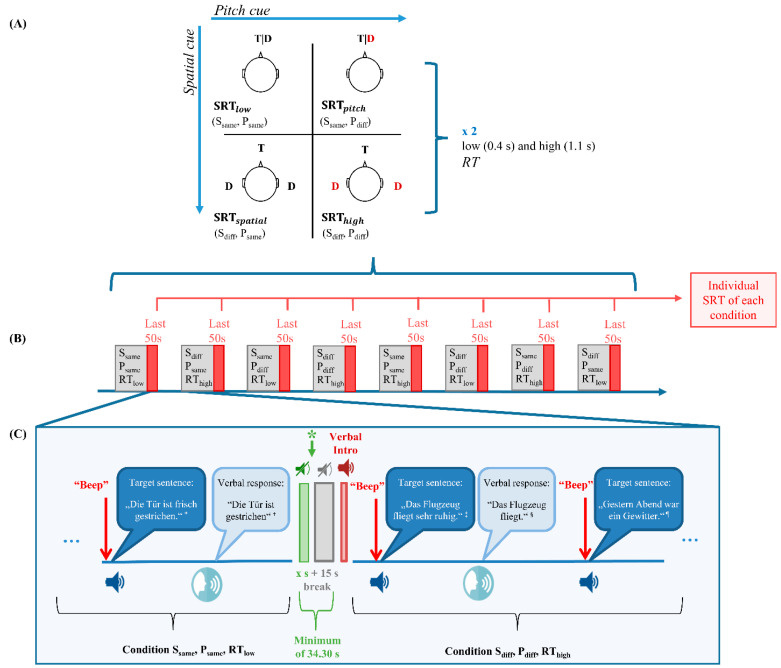
Illustration of the eight testing conditions and the procedure. (**A**) The 2 pitch cues × 2 spatial cues × 2 reverberation times design. Illustration adapted with permission from Peng, Pausch, and Fels [105]. (**B**) Example of all eight testing conditions with the last 50 s being of relevance for the neural analyses. (**C**) Excerpts of the testing procedure showing a 200 ms long beep and subsequent 500 ms silence that preceded each target sentence (see the Supporting Material for exemplary audio files and task instructions) as well as the verbal response. In between conditions, a playback introduced the next condition and a manually initiated (asterisk) break with a total silence duration of at least 30.4 s was presented before each condition. Abbreviations: SRT—speech reception threshold; T—target voice; D—distractor voices; S_same_—target and distractor at the same spatial position in front of participant; S_diff_—target at front and distractors at ± 90°; P_same_—same pitch of target and distractor voices (both “D” and “T” in black color); P_diff_—different pitch of target and distractor voices (“T” in black and “D” in red); RT_low_—low reverberation time; RT_high_—high reverberation time. Footnotes: * The door is freshly painted. † The door is painted. ‡ The plane flies very quietly. § The plane flies. ¶ Last night was a thunderstorm.

In the VAE, a modified SIN task (adjusted from the LISN-S task by Cameron and Dillon [27]) for the assessment of German-speaking children was implemented [105]. The target speech was a selection of 5-keyword sentences from the Hochmair-Schulz-Moser (HSM) test [106] and recorded with a native German-speaking female voice (mean f_0_ = 213 Hz, measured from a 2-min utterance). The two-talker distractor speech was passages from fairy tales by the Grimm brothers that children were less familiar with. Two pitch conditions were created by either using the voice from the target for the distractors (P_same_), or two separate female voices (P_diff;_ mean f_0_ = 191 and 198 Hz). In the VAE, the target speaker was always positioned in front of the listener. To introduce spatial separation, the distractor speakers were either located symmetrically on both sides of the participant with a 90° angular separation (S_diff_) or in the same virtual position as the target speaker (S_same_). The 2 spatial × 2 pitch conditions were tested in a low reverberant virtual classroom (0.4 s with reverberation time (RT), averaged across octave bands between 500 and 2000 Hz; RT_low_) and a high reverberant virtual classroom (1.1 s; RT_high_), which were created through variations in absorption and scattering properties of the surface materials of the virtual classroom. Thus, eight conditions, one test block each, were created through variations of pitch and spatial cue, and RT (Figure 4A,B). The order of the test blocks was pseudorandomized following a nested Latin Square design.

For each child, a short practice run was provided for task familiarization. During the main task, a manually initiated 15 s rest period, with a subsequent audio playback introducing the next condition, was presented prior to each block (Figure 4B,C). Note that due to the manual start of the rest block, which allowed each child to determine individually when they were ready to continue to account for fatigue, the total duration of rest was variable (*M* = 44.52; *SD* = 17.81; minimum of 34.30 s, allowing the fNIRS signal to return to baseline). At the beginning of each condition, the distractor stories started and continued throughout the entire test block. A leading 1 kHz sine tone of 200 ms was played, followed by 500 ms silence, before each target sentence was presented. The child verbally repeated what was heard. The verbal response was manually scored based on the accurately identified keywords by an experimenter outside the booth. An excerpt of the procedure is shown in Figure 4C.

The speech reception threshold (SRT) is a measure of speech comprehension in noise, with lower values indicating better behavioral performance. For each condition/block, the SRT at 50% accuracy was tracked using a one-down one-up adaptive staircase procedure [107], adjusting the target presentation level at an initial step size of 4 dB. The distractors were always presented at 55 A-weighted decibels (dBA) sound pressure level (SPL). The target speech was set at an initial 70 dBA SPL. A trial was scored correct with three or more keywords correctly verbally repeated. This led to the subsequent trial with lowered target speech, i.e., lower SNR, until the first reversal, and then, step size subsequently changed to 2 dB. A reversal was reached when the direction of changing SNR reversed, such as from decreasing to increasing SNR. A test block terminated at the 6th reversal. To ensure safety, the playback levels never exceeded 80 dBA SPL for children with NH and 105 dBA SPL for the child using the research HAs.

### 2.4. Preprocessing

#### 2.4.1. Behavioral Data

To derive an SRT for each acoustic condition, a logistic regression was fitted to all SNRs tested with the SNR at 50% accuracy being interpolated [108]. For children, this approach is considered more robust [109] and consistent in estimating the psychophysical threshold from disperse behavioral data [110], as compared to SRTs calculated by averaging the last reversals.

#### 2.4.2. Neural Data

Appertaining to previous findings [54,56,88], the bilateral STG and IFG formed our 4 a priori hypothesized ROIs. CHs assessing bilateral IFG and STG activity are depicted in Figure 3A. Inclusion of a participant required at least 50% of CHs in each ROI to have a good signal quality. As the usage of an automated, criterion-based detection of bad signal quality might sometimes be obstructed due to large baseline shifts and trends in the data, particularly in paradigms of long duration, poor signal quality was identified by visual inspection before and after preprocessing (i.e., CHs showing a large signal variation and spikes or measurement errors/flat lines). On average, 1–2 CHs across all ROIs and probe sets were excluded in the remaining sample.

The fNIRS data were preprocessed in MATLAB [98] via self-written scripts and scripts from the HomER2 (Huppert et al. [111]; version: homer2_src_v2_8_11022018) and SPM-fNIRS toolbox [112]. All steps of the preprocessing pipeline are depicted in Figure 3B. Specifically, a combined spline interpolation and wavelet filtering approach was used to reduce motion artifacts, because the combination of the two techniques yielded the best results for data obtained in challenging samples and tasks as well as for paradigms that involve motion [113,114]. Further, the last 50 s of each block were considered for the neuronal analyses (Figure 4C), because the last 50 s best captured the neuronal activation of the SRT of each individual, i.e., the last 4 reversals comprising the last 5–6 sentences per condition. During this time, the SNR was reached, at which the child heard 50% of the sentences correctly.

### 2.5. Analyses

Statistical analyses for the pilot data were performed in IBM SPSS [115] and R [116]. For the NH group, a repeated measures analysis of variance (rmANOVA) was fitted to the behavioral measure of SRTs and neural activity (ΔHbT) in each ROI, separately, using 3 within-subject factors: RT (RT_low_ vs. RT_high_), spatial (S_same_ vs. S_diff_), and pitch cue (P_same_ vs. P_diff_). An a priori α = 0.05 was used to identify statistical significance. For post hoc analysis using pairwise comparisons, uncorrected *t*-test results are reported because of the small sample size in this pilot study and the main purpose of applicability assessment.

To examine behavioral SRTs during HAs use, exploratory analyses were conducted in an available program (SINGLIMS.EXE; Crawford et al. [117], Crawford and Garthwaite [118]) using modified *t*-tests [119]. That is, individual data of children with HAs were compared against the group distribution estimated from the NH group. For further inspection, the effect size with 95% confidence intervals and a point estimate of the probability of a HA user’s score falling above the value of the NH group are presented. For the neural data, effects on ΔHbT that were identified in the NH group analysis were similarly plotted separately for each child of the HA group for exploratory purposes. Importantly, the results are primarily intended to demonstrate applicability in children fitted with HAs rather than generalizable evidence across the population that is hard of hearing and fitted with HAs.

## 3. Results

### 3.1. Behavioral Data

**Figure 5:**
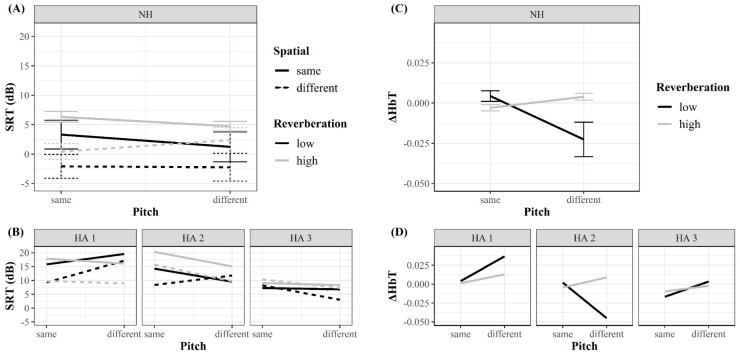
Effects of reverberation time, speech, and pitch on the speech reception threshold (SRT) and total hemoglobin concentration changes (ΔHbT) in the left inferior frontal gyrus (IFG). (**A**) SRT plotted as a function of pitch cues as group mean for children with normal hearing (NH). (**B**) Individual SRT data for the three children in the hearing aid (HA) group. (**C**) Effect of reverberation time and pitch on ΔHbT in the left IFG of the NH group. (**D**) Individual ΔHbT for the three children in the HA group. Error bars (**A** and **C**) represent the standard error. Line color indicates reverberation time (high in gray, low in black). Line style indicates spatial cue availability (same spatial position (straight line) or different position (dotted line)).

Figure 5A illustrates average SRTs from the NH group in each test condition. For the NH group, the rmANOVA of SRTs at 50% accuracy revealed a significant main effect of RT, *F*(1,2) = 12.81, *p* = 0.02, η_p_^2^ = 0.72. When collapsed across spatial–pitch conditions, speech comprehension improved on average by 3.41 dB in SRT when RT reduced from 1.1 to 0.4 s. Although neither the main effect of spatial cue (*p* = 0.05) nor pitch cue was statistically significant (*p* = 0.18), there was a significant interaction between these two factors *F*(1,5) = 19.56, *p* = 0.007, η_p_^2^ = 0.80. When averaged across RTs, the comprehension benefit from the 90° spatial separation was significantly larger in the same pitch condition (*M* = 5.64 dB) than in the different pitch condition (*M* = 2.91 dB), *t*(5) = 3.48, *p* = 0.02, *d* = 1.42. No other comparisons were found to be significant.

For children with HAs, the individual SRT (Figure 5B) was compared to the NH group in each test condition using modified *t*-tests [119]. Large individual variability was observed (Table 2). In general, most children of the HA compared to the NH group had elevated SRT in all eight test conditions. Using the SRT distributions from the NH group, the probability of the children with HAs’ score falling above the value of the NH group ranged between 81.75 and 99.89% for child HA 1, between 78.26 and 99.84% for child HA 2, and between 57.21 and 97.90% for child HA 3 across all eight conditions. Among all children with HAs, child HA 3, with the best unaided thresholds in the pure tone audiogram, had SRTs closest to the NH group.

### 3.2. Neural Data

The repeated-measures ANOVA of the ∆HbT for the NH group revealed a significant interaction between the pitch cue and RT in the left IFG, *F* (1,5) = 7.38, *p* = 0.04, η_p_^2^ = 0.60. Irrespective of spatial cue availability, left IFG activation tended to be lower during the condition with low RT and available pitch cue (RT_low,_ P_diff_) than in conditions with high RT and available pitch cue (RT_high,_ P_diff_; Figure 5C; *t*(5) = 2.42, *p* = 0.06, *d* = 1.18). Similar activations in the left IFG were observed between the two RTs when the target and masker shared the same pitch. Results suggested that the pitch cue reduced the LIFG activation only in the low RT but not in high RT. No other effects on ∆HbT, or other analyses for the remaining ROIs, were found to be significant.

To explore possible alterations, the activation of each child in the HA group was plotted for the interaction effect observed in the NH group (Figure 5D). Notably, child HA 3, with the best unaided pure tone audiogram and best behavioral performance, showed different activation patterns than the NH group. On the contrary, child HA 2 showed neural activation patterns similar to the NH group, although the behavioral performance of child HA 2 was poorer.

## 4. Discussion

The main objective of the current pilot study was to provide a tentative assessment of applicability and to offer extensive recommendations for future applications of a novel paradigm and experimental setup that combines fNIRS and VAEs to investigate simulated complex real-world listening in children on behavioral and neural levels. Furthermore, the multimethod approach was tested in three children with bilateral HAs.

The findings of the pilot study suggest that excessive reverberation of 1.1 s impairs speech comprehension in children with NH on the behavioral level. This corroborates with previous reports of the negatively affected hearing and wellbeing of children in reverberating classrooms [120,121]. According to the American Speech–Language–Hearing Association guidelines for classroom recommendations, good classroom acoustics should be controlled to be under 0.6 s and between 0.45 and 0.6 s according to European regulatory guidelines [122]. Next to the room acoustics, the spatial separation between the target and distractors might provide important auditory cues for children with NH to understand SIN. In the behavioral measure of SRT, the effect of spatial cues was moderated by pitch similarity between the target and distractors in line with Cameron and Dillon [27]. The NH group received a larger speech comprehension benefit from the spatial separation when the target and distractors shared the same pitch. This might suggest that pitch similarity promoted the use of spatial cues for understanding SIN. Behavioral performance of the HA group was overall poorer across conditions. Nevertheless, most children fitted with HAs also benefited from the spatial separation of the talkers. There was large individual variability in the effect of RT and pitch cue on performance. The degree of HL likely accounted for the variance in performance. Indeed, better aided speech audibility as well as stronger vocabulary and working memory abilities have been shown to facilitate SIN recognition in reverberant environments [24]. Additional studies applying the multimethod approach are required to clarify the observed variability and its underlying mechanisms.

Crucially, while the current study pointed to the potential of the novel multimethod approach to investigate complex, realistic listening scenarios in NH children and children with HAs, the current fNIRS results in particular should be treated with caution due to the small sample size and the exploratory nature of the pilot study. On the neural level, in the low RT condition, a reduced left IFG activation was observed by introducing the pitch cue. This might suggest that the left IFG assists SIN recognition during more difficult, effortful conditions only (i.e., when target and distractor talkers share the same pitch) to reach a comparable behavioral performance to easier listening conditions (i.e., when a pitch cue is introduced). This finding corroborated with previous studies that considered attention-dependent left IFG activation a plausible neural marker for effortful listening [55,88,123]. While the behavioral measure of SRTs improved by the introduction of the pitch cue only if target and distractors shared the same spatial position, the neural finding might indicate that children received a general release from effortful listening by the pitch cue. Interestingly, the release from effort was only accessible by children in low RT but not in high RT. This might suggest that the high RT conditions require more effort irrespective of pitch cue availability. In contrast, no differences in STG activation were observed between test conditions. Analogously to the behavioral performance, a large variety of the effect of RT and pitch cues on neural activation in the left IFG was observed for the children with HAs. Of note, HA user 3, who had the best behavioral performance and the best unaided pure tone audiometry, did not show similar activation patterns to the NH group. HL has been shown to lead to neural alterations within and beyond the auditory cortices [124,125]. Different neural resources might support speech comprehension in complex auditory conditions in children with bilateral HL and HAs compared to children with NH. Yet, future studies with a modified VAE-fNIRS approach and a larger sample size have yet to identify the exact neural mechanisms within and beyond bilateral STG and IFG that support speech comprehension after HL and HA use.

While fNIRS hyperscanning alone already allows to capture neural activation of two people during natural conversations [66,126], the fNIRS-VAE approach could potentially enable the investigation of even more complex auditory situations in the long-run. By simulation of multiple speakers as well as varying acoustic room conditions, each factor that contributes to real-world hearing could be investigated in isolation and related to underlying neural mechanisms. It should be noted that real-life auditory perception generally takes place in a multisensory environment. While the role of tactile cues is still unclear, visual cues were shown to affect auditory perception in (virtual) environments [127,128]. Importantly, visual cues improve SIN perception differently in children with HL compared to children with NH, with a larger audio–visual enhancement for children with HL [129]. Thus, while it is possible that additional visual cues may have been particularly helpful for the children with HAs, the current design focused on firstly understanding the auditory aspect through highly controllable VAEs. Nevertheless, future research studies incorporating other cues in the virtual environment, such as visual cues, could be of interest in the long run. Further, while most research utilizing traditional SIN paradigms is interested in performance differences (speech comprehension) and associated varying effort levels, for example between the NH population and individuals with HL, the staircase procedure offers the exploitation of different research questions. Constant accuracy levels between participants and conditions might enable the investigation of behavioral and neural mechanisms that facilitate such a comparable level of speech comprehension in children with NH as well as children with HAs. In addition to classical audiometric testing in simple acoustic environments, advanced fNIRS-VAE approaches might, after extensive validation, potentially also offer the possibility to optimize HA fitting in complex auditory scenes and to identify possible factors that could be improved in assistive listening devices.

Despite its numerous advantages, future research using a modified VAE-fNIRS application is warranted to validate the current findings and to further elucidate the behavioral and neural mechanisms that underlie individual differences in SIN comprehension in children with NH and children fitted with HAs. In order to benefit from the current VAE-fNIRS pilot study in future applications of the multimethod approach, an extensive list of several limitations of the presented approach and recommendations of how to address each of the challenges for future multimethod applications are offered in Table 3.

In conclusion, while several challenges are still to be overcome and future studies have to further evaluate adapted versions of this multimethod approach, the application of advanced VAE-fNIRS approaches could provide unique tools to understand children’s listening abilities in complex real-world auditory situations and potentially offer crucial information to improve assistive fitting of HAs in complex (simulated) real-world listening in the long-run.

## Figures and Tables

**Table 1:** Examples of SIN tests and investigations.

	Outcome Measure	Population of Interest	Behavioral/Neuroimaging Method	Test/Example Studies	Overview
**A**	**Behavioral SIN Assessments**				
	SIN recognition assessments with varying SNR and/or noise location	Adults with NH/HL	Behavioral	Hearing In Noise Test—HINT [25]	Headphone-based; recordings of 250 sentences by a male speaker that are intended to be utilized in adaptive SRT measurements in quiet or spectrally matched noise
		Adults with NH/HL		Oldenburger Satztest (Oldenburger sentence test)—OlSa [28,29]	Headphone-based; recordings of sentences that consist of a random combination of 50 words that are used to measure the SRT in quiet and in noise
		Adults with NH/HL		Words-In-Noise test—WiN [26], for clinical use	Earphone-based; recordings of 70 words embedded in unique segments of multi-talker distractor noise that are intended to be utilized in adaptive SRT measurements
		Adults with NH/HL		Döring test [30], for clinical use	Loudspeaker-based; recordings are single syllables of the “Freiburger Sprachverständnistest” (Freiburger speech comprehension test) which are repeated three times in background noise (words of the “Freiburger Sprachverständnistest”); spatial location of noise and target are varied (spatially separated vs. co-located)
		Children with NH/HL		Listening in Spatialized Noise-Sentences test—LiSN-S [27]	Headphone-based; recordings of 120 sentences by a female speaker that are intended to be utilized in adaptive SRT measurements in background speech by two masking talkers (two female speakers that record two distractor stories) in four different conditions: maskers are either spatially co-located with target or at ±90° azimuth and either share the same pitch or different pitch than the target
		Children with NH/HL		“Oldenburger Kinder-Satztest” (Oldenburger sentence test for children)—OlKiSa [31]	Headphone-based; simplified version of the Oldenburger sentence test (OlSA); recordings of sentences that consist of a random combination of 21 words that are used to measure the SRT in quiet and in noise
		Children with NH/HL		Children’s Coordinate Response Measure—CCRM [32]	Headphone-based; recordings of sentences that are to be utilized in adaptive SRT measurements in either 20-talker babble or speech-shaped noise
**B**	**Listening in the Free Field or VAEs**				
	Sound localization	Adults with NH	Behavioral	Bronkhorst [33], Wenzel et al. [34], Denk et al. [35], Pausch and Fels [36]	Investigations of auditory sound localization, distance perception, and attention switching using ear/headphones, research HAs, or loudspeaker-based reproduction of auditory stimuli with or without manipulation of acoustic variables including but not limited to reverberation, interaural level differences, and sound intensity
		Adults with HL		Best et al. [37], van den Bogaert et al. [38]	
		Children with HL		Johnstone et al. [39]	
	Auditory distance perception	Blind and sighted adults with NH		Kolarik et al. [40], Kolarik et al. [41]; Shinn-Cunningham [42], Zahorik [43]	
		Adults with NH/HL		Courtois et al. [44]	
	Auditory attention switching	Adults with NH		Oberem et al. [45], Oberem et al. [46]	
	Auditory simulations of SIN tasks in simulated indoor environments	Adults with NH	Behavioral	MacCutcheon et al. [47], Peng and Wang [48,49], Helms Tillery et al. [50]	Investigations of speech or word (in noise) recognition, listening effort, and the influence of variables such as language skills, working memory, or stimulus presentation, i.e., auditory-only or in combination with visual stimuli, and room acoustics such as reverberation times simulated VAEs
		Children with HAs/NH		McCreery, Walker, Spratford, Lewis, and Brennan [24]	
		Children with NH		Rudner, Lyberg-Ahlander, Brannstrom, Nirme, Pichora-Fuller, and Sahlen [13], MacCutcheon et al. [51], MacCutcheon et al. [52]	
		Adults with NH/HAs		Ricketts et al. [53]	
**C**	**Speech Comprehension Neuroimaging Studies**				
	Speech/SIN recognition, the effect of cochlear implantation, age-related or early onset HL, and the underlying neural mechanisms that are identified by invasive (i.e., ECoG) or noninvasive (i.e., EEF, fNIRS, fMRI) neuroimaging	Adults with NH	fNIRS	Defenderfer et al. [54], Wijayasiri et al. [55], Zhang et al. [56]	Investigations of (selective attention to) speech or word in quiet or noise recognition and their underlying neural mechanisms, by means of spatial and/or temporal neural analyses, using head/earphone- or free-field loudspeaker-based auditory reproduction while manipulating auditory (and visual) stimulation or using real-life hyperscanning (i.e., measuring two or more participants at the same time) paradigms
		Adults with NH/CI	fNIRS	Olds et al. [57], Rowland et al. [58], Anderson et al. [59]	
		Children with NH	fNIRS	Mushtaq et al. [60]	
		Children with CI	fNIRS	Mushtaq et al. [61]	
		Adults with age-related HL	EEG	Puschmann et al. [62], Marsella et al. [63]	
		Infants	EEG-fNIRS	Telkemeyer et al. [64]	
		Adults with NH	fNIRS hyperscanning	Dai et al. [65], Jiang et al. [66]	
		Adults with medically intractable epilepsy	ECoG	Zion Golumbic et al. [67]	
		Adults with NH	EEG-fMRI	Puschmann et al. [68]	
		Adults with age-related HL	fMRI	Wong et al. [69]	

Abbreviations: SIN—speech-in-noise; SRT—speech reception threshold; VAE(s)—virtual acoustic environment(s); SNR—signal-to-noise ratio; NH—normal hearing; HL—hearing loss; CI—cochlear implant; HA—hearing aid; fNIRS—functional near-infrared spectroscopy; EEG—electroencephalography; ERP—event-related potential; ECoG—intracranial electrocorticograph; fMRI—functional magnetic resonance imaging.

**Table 2:** Comparison of SRTs from individual children with bilateral HAs with the control group of children with NH.

Condition	Statistics	HA 1	HA 2	HA 3
**RT_low_S_same_P_same_**	*t*-value	1.93	1.70	0.62
	two-tailed probability	0.11	0.15	0.57
	Effect size and 95% CI	2.08 (0.58–3.55)	1.84 (0.45–3.17)	0.66 (−0.26–1.53)
	Estimated percentage of HA case falling above NH group (CI)	94.42 (71.79–99.98)	92.51 (67.30–99.92)	71.72 (39.90–93.74)
**RT_low_S_same_P_diff_**	*t*-value	2.73	1.24	0.83
	two-tailed probability	0.04 *	0.27	0.45
	Effect size and 95% CI	2.95 (1.00–4.87)	1.34 (0.17–2.45)	0.89 (−0.10–1.83)
	Estimated percentage of HA case falling above NH group (CI)	97.93 (84.02–100)	86.50 (56.92–99.28)	77.73 (46.05–96.64)
**RT_low_S_diff_P_same_**	*t*-value	2.14	1.97	1.94
	two-tailed probability	0.09	0.11	0.11
	Effect size and 95% CI	2.31 (0.69–3.89)	2.12 (0.60–3.61)	2.09 (0.58–3.56)
	Estimated percentage of HA case falling above NH group (CI)	95.74 (75.53–100)	94.68 (72.47–99.98)	94.48 (71.95–99.98)
**RT_low_S_diff_P_same_**	*t*-value	3.12	2.26	0.85
	two-tailed probability	0.03 *	0.07	0.43
	Effect size and 95% CI	3.37 (1.19–5.54)	2.44 (0.76–4.10)	0.92 (−0.08–1.86)
	Estimated percentage of HA case falling above NH group (CI)	98.69 (88.35–100)	96.35 (77.52–100)	78.30 (46.67–96.87)
**RT_high_S_same_P_same_**	*t*-value	4.72	5.74	1.12
	two-tailed probability	0.005 **	0.002 **	0.31
	Effect size and 95% CI	5.10 (1.96–8.26)	6.20 (2.42–10.00)	1.21 (0.10–2.26)
	Estimated percentage of HA case falling above NH group (CI)	99.74 (97.47–100)	99.89 (99.23–100)	84.32 (53.90–98.81)
**RT_high_S_same_P_diff_**	*t*-value	5.20	4.73	1.66
	two-tailed probability	0.003 **	0.005 **	0.16
	Effect size and 95% CI	5.61 (2.18–9.07)	5.11 (1.96–8.28)	1.79 (0.42–3.11)
	Estimated percentage of HA case falling above NH group (CI)	99.83 (98.52–100)	99.74 (97.50–100)	92.09 (66.42–99.90)
**RT_high_S_diff_P_same_**	*t*-value	2.63	4.28	2.81
	two-tailed probability	0.047 *	0.008 **	0.04 *
	Effect size and 95% CI	2.84 (0.94–4.70)	4.62 (1.75–7.50)	3.03 (1.04–5.00)
	Estimated percentage of HA case falling above NH group (CI)	97.66 (82.73–100)	99.61% (95.97–100)	98.11 (84.98–100)
**RT_high_S_diff_P_diff_**	*t*-value	1.19	1.33	0.89
	two-tailed probability	0.29	0.24	0.41
	Effect size and 95% CI	1.28 (0.14–2.36)	1.44 (0.23–2.59)	0.96 (−0.06–1.92)
	Estimated percentage of HA case falling above NH group (CI)	85.58 (55.61–99.10)	87.98 (59.13–99.52)	79.30 (47.79–97.26)

Modified *t*-test statistics performed based on methods described by Crawford and Howell [119] are listed for each child in the HA group, by comparing the individual’s speech reception threshold to the NH group in each test condition. Effect sizes with 95% confidence interval are shown between the individual case and control group. Abbreviations: HAs—hearing aids; NH—normal hearing; RT_low_—low reverberation time; RT_high_—high reverberation time; S_same_—same spatial position of target and distractor speakers; S_diff_—different position of target and distractor speakers; P_same_—same pitch of target and distractor voices; P_diff_ different pitch of target and distractor voices; CI—confidence interval; M—mean; SD—standard deviation; * *p* < 0.05; ** *p* < 0.01.

**Table 3:** Challenges and recommendation for future combined fNIRS and VAEs applications.

Aspect	Challenge(s)	Explanation	Considerations/Recommendations to Address Challenges
**Task**	Long task duration and long-lasting fNIRS cap wearing	Lengthy and strenuous paradigm for younger children (~30 min; especially long duration if the speech recognition in background noise is good due to staircase procedure)	Administer the task in **several testing sessions/days** when possible. Focus on **fewer variables of interest** that might affect listening in background noise.
**FNIRS measurement during adapted versions of the current task design**	Repetition of task conditions	For fNIRS measurements, ideally, repetitions of testing conditions within each subject are warranted; currently, each condition is only presented once	**Increase repetitions of test conditions**, e.g., by several testing sessions (see recommendations for task design), to diminish the effect of noise in the fNIRS signal and measurement errors.
	Disentangling behavioral performance and manual presentation of target sentences	Currently, listening and speaking are both included in the mean concentration changes of HbT; manual presentation times of the target sentence led to differing combinations of target-distractor speech	**Event-related design** with fewer conditions or **block design** with **fixed presentation times** (i.e., fixed time periods for the occurrence of events) should be considered. For an overview of advantages and disadvantages of block- and event-related fNIRS designs in auditory assessments, see van de Rijt et al. [130].
	Perceived vs. (physical) loudness intensity	The amplitude of the fNIRS signal might be affected by sound intensity.	Loudness deviations when investigating SIN comprehension typically do not exceed 10 dB SPL. Activation differences thus hardly reflect overall sound intensity differences. Nevertheless, individual loudness perception (rather than physical intensity) appears to be related to brain activation [130,131,132] and **subjective auditory loudness perception should be assessed** and taken into consideration during interpretation.
	Noise removal: Head movements and high pass filtering	Head movements are warranted during VAE simulations; however, an excessive amount might distort the NIRS signal. The long duration of the task limits the strict application of high pass filters.	For datasets that are acquired from challenging samples, few trials, lengthy paradigms, and when head movements are an important aspect of the task, **combined motion artifact detection and correction techniques** are highly recommended (e.g., see Jahani, Setarehdan, Boas, and Yucel [113] or Di Lorenzo, Pirazzoli, Blasi, Bulgarelli, Hakuno, Minagawa, and Brigadoi [114]). **Implementation of short-separation CHs**, which are sensitive to changes in superficial blood flow, is considered crucial to remove noise (i.e., extra-cerebral signal) [133,134], which is also highly relevant due to the long task duration that limits the application of strict high pass filters. When investigating various age groups, an **age-corrected differential path length factor** is advised [135].
	Speech-induced motion artifacts	Chin clasps of the cap might transfer speech-induced motion of the jar	Usage of (EEG) caps to ensure **firm hold without usage of chin clasp**. An attachment of the ends of the cap to the upper body should be considered. **Fixate fiber bundles** (if no **wireless device** available) to the fNIRS cap to prevent movements being transmitted from fiber bundles to optodes.
	Localization/ROIs and lateralization	Variability in head size and shape might affect the formation of ROIs and a differential lateralization of speech-related activity might add additional variation.	The use of **probe positioning units** ensures correct and consistent fNIRS probe placement. Individual formation of ROIs **by allocation of relative weights to the CHs depending on the probability to fall into a respective ROI** (e.g., see Huppert et al. [136]) might be considered for the analyses. In addition, **variability in speech lateralization** due to inter alia speech content [137,138,139] should be controlled for.
**Participants**	Varying degrees of HL, HA devices, and frequency of HA use	Due to time constraints and elaborative purpose of the study design, an audiometry was performed only for the HA group that served as input for the research HAs	Future studies, assessing larger populations, should aim at **controlling for varying degrees of hearing (loss)** and administer detailed questionnaires about **HA use, device**, **and fitting**.
	Other factors affecting speech comprehension	Due to the small sample size, the current pilot investigation could not control for variability in hearing abilities. Auditory, linguistic as well as other cognitive mechanisms were suggested to affect speech understanding (e.g., see the ease of language understanding model Rönnberg, Lunner, Zekveld, Sörqvist, Danielsson, Lyxell, Dahlström, Signoret, Stenfelt, and Pichora-Fuller [19], Rönnberg, Holmer, and Rudner [20], Holmer, Heimann, and Rudner [21] or McCreery, Walker, Spratford, Lewis, and Brennan [24]). Speech represents a highly complex auditory signal that involves multiple brain networks. Animal models of cortical reorganization following HL highlight the widespread effects on HL beyond the auditory cortex and the interplay of multiple neural networks that, in turn, make the effects of HL on speech understanding highly individual [98,99].	Next to audiometry, **additional measures on cognition and speech performance** (e.g., assessment of (verbal) IQ and speech production) were beyond the scope of the current pilot study; however, they are highly recommended for future applications.

Abbreviations: fNIRS—functional near-infrared spectroscopy; VAE(s)—virtual acoustic environment(s); HbT—total hemoglobin; CHs—channels; EEG—electroencephalography; ROI(s)—region of interest(s); HA—hearing aid; HL—hearing loss.

## Supplementary Materials

The following are available online at https://www.mdpi.com/2227-9067/7/11/219/s1, Supplementary Table S1: An overview of the study material and equipment; Supplementary Table S2: Items of demographic questionnaire and hearing aid assessment; Supplementary Audio Files (.mp3): 16 exemplary audio files that include the playback of the 8 test conditions for the children with normal hearing and the playback of the 8 test conditions for the children with hearing loss.
