# Supplementary material for: fNIRS Assessment of Speech Comprehension in Children with Normal Hearing and Children with Hearing Aids in Virtual Acoustic Environments: Pilot Data and Practical Recommendations
Source: Children (Basel). 2020 Nov 7;7(11):219. doi: 10.3390/children7110219 (PMC7695031; doi:10.3390/children7110219)
Supplement: Supplementary file 1 [file children-07-00219-s001.zip › SuppFile_DemographicData.pdf]

Manuscript: fNIRS assessment of speech comprehension in children with normal hearing and children with hearing aids in virtual acoustic environments: Pilot data and practical recommendations

S2: Items of demographic questionnaire and hearing aid assessment

**A.**	**Demographic information**
	a. Date of birth<br>b. Gender<br>c. List of all psychiatric and neurological conditions of the child<br>d. List of all prescribed drugs that the child is currently taking
**B.**	**Information on hearing aid (HA) usage**
	a. Month/Year in which the child first received the first HA<br>b. Month/Year in which the last HA fitting took place<br>c. Hours the HAs are worn during a school day<br>d. Hours the HAs are worn during a school out of school
**C.**	**Pure-tone audiometry**
