# Supplementary material for: fNIRS Assessment of Speech Comprehension in Children with Normal Hearing and Children with Hearing Aids in Virtual Acoustic Environments: Pilot Data and Practical Recommendations
Source: Children (Basel). 2020 Nov 7;7(11):219. doi: 10.3390/children7110219 (PMC7695031; doi:10.3390/children7110219)
Supplement: Supplementary file 1 [file children-07-00219-s001.zip › SuppFile_Legend.pdf]

Manuscript: fNIRS assessment of speech comprehension in children with normal hearing and children with hearing aids in virtual acoustic environments: Pilot data and practical recommendations

S1: An overview of the study material and equipment

RESOURCE	DESCRIPTION/ SOURCE	FORMAT
**Data**		
Questionnaire items	Supporting information:<br>SuppFile_DemographicData.docx	Text file (.doc)
Target speech stimuli	Current paper; HSM Test:<br>Hochmair-Desoyer, I., Schulz, E., Moser, L., & Schmidt, M. (1997). The HSM sentence test as a tool for evaluating the speech understanding in noise of cochlear implant users. *Otology & Neurotology*, *18*(6), S83-S86.	Text file (.doc)/ Article
Exemplary speech material	Supporting information:<br>Separate files for the children with normal hearing (NH) and for children with hearing loss (HL):<br><br>A separate file for each of the 8 test conditions:<br>SuppFile_1_Ssame_Psame_Rlow_NH.mp3<br>SuppFile_2_Ssame_Psame_Rhigh _NH. mp3<br>SuppFile_3_Ssame_Pdiff_Rlow _NH. mp3<br>SuppFile_4_Ssame_Pdiff_Rhigh _NH. mp3<br>SuppFile_5_Sdiff_Pdiff_Rlow _NH. mp3<br>SuppFile_6_Sdiff_Pdiff_Rhigh _NH. mp3<br>SuppFile_7_Sdiff_Psame_Rhigh _NH. mp3<br>SuppFile_8_Sdiff_Psame_Rlow _NH. mp3<br><br>SuppFile_1_Ssame_Psame_Rlow_HL. mp3<br>SuppFile_2_Ssame_Psame_Rhigh _HL. mp3<br>SuppFile_3_Ssame_Pdiff_Rlow _HL. mp3<br>SuppFile_4_Ssame_Pdiff_Rhigh _HL. mp3<br>SuppFile_5_Sdiff_Pdiff_Rlow _HL. mp3<br>SuppFile_6_Sdiff_Pdiff_Rhigh _HL. mp3<br>SuppFile_7_Sdiff_Psame_Rhigh _HL. mp3<br>SuppFile_8_Sdiff_Psame_Rlow _HL. mp3	.mp3
**Reproduction Hardware, Laboratory Equipment**		
Intel Core i7, 8 GB RAM	Intel Corporation, Santa Clara, California, United States	Personal Computer
RME Fireface UC	Audio AG, Haimhausen, Germany	Audio Interface
4x Neumann KH 120A	Georg Neumann GmbH, Berlin, Germany	Loudspeakers
2x Research Hearing Aids / Cables	GN ReSound, Ballerup, Denmark / Sonion, Roskilde, Denmark	Hearing Aid Prototype

NTG 2	Røde Microphones, Silverwater, Sydney, Australia	Participant Microphone
HD 650	Sennheiser electronic GmbH & Co. KG, Wedemark, Germany	Experimenter Headphones
K&M 29375 / CM 20P	König & Meyer GmbH & Co. KG, Wertheim am Main, Germany / Samson Technologies, Hauppauge, New York, United States	Experimenter Feedback Table Microphone
C920	Logitech international S.A., Lausanne, Switzerland	Supervision Webcam
4x Flex 13, OptiHub2, CS200 / CW-500, Rigid Body Base, Marker Set	OptiTrack, NaturalPoint, Inc., Corvallis, Oregon, United States	Optical Infrared Tracking System
**fNIRS device**		
ETG-4000	Hitachi Medical Corporation, Tokyo, Japan<br>2x3x5 optode configuration<br>(see current manuscript for description of placement)	Optical imaging device
fNIRS cap	Easycap GmbH, Herrsching, Germany	Cap
**Preprocessing and Analysis Software**		
MATLAB	The MathWorks Inc.	Software
SPM for fNIRS	Tak, S., Uga, M., Flandin, G., Dan, I., & Penny, W. D. (2016). Sensor space group analysis for fNIRS data. *J Neurosci Methods*, *264*, 103-112. doi:10.1016/j.jneumeth.2016.03.003 (Software retrieved from https://www.nitrc.org/projects/spm_fnirs/)	Article/ HTML link
HomER2	Huppert, T. J., Diamond, S. G., Franceschini, M. A., & Boas, D. A. (2009). HomER: a review of time-series analysis methods for near-infrared spectroscopy of the brain. Applied optics, 48(10), D280-D298. doi: 10.1364/AO.48.00D280; (version: homer2_src_v2_8_11022018, software retrieved from https://www.nitrc.org/projects/homer2/)	Article/ HTML link

ITA-Toolbox	Berzborn, M., Bomhardt, R., Klein, J., Richter, J.G., & Vorländer, M. (2017). The ITA-Toolbox: An Open Source MATLAB Toolbox for Acoustic Measurements and Signal Processing. In *43th Annual German Congress on Acoustics, Kiel (Germany), 6 Mar 2017 - 9 Mar 2017*. (Software retrieved from http://www.ita-toolbox.org/)	Software / URL
Virtual Acoustics (VA)	Institute of Technical Acoustics, RWTH Aachen University. Virtual Acoustics – A real-time auralization framework for scientific research. (Software retrieved from http://www.virtualacoustics.org)	Software / URL
Room Acoustics for Virtual Environments (RAVEN)	Schröder, D. (2011). *Physically based real-time auralization of interactive virtual 851 environments* (Vol. 11): Logos Verlag Berlin GmbH.;	Article
Motive	OptiTrack, NaturalPoint, Inc., Corvallis, Oregon, United States (Software https://optitrack.com/products/motive/)	Software / URL
Master Hearing Aid (MHA)	HörTech gGmbH, Oldenburg, Germany / Grimm, G., Herzke, T., Berg, D., & Hohmann, V. (2006). The master hearing aid: A PC-based platform for algorithm development and evaluation. *Acta acustica united with Acustica, 92*(4), 618-628. (Retrieved from https://www.ingentaconnect.com/content/dav/aaua/2006/00000092/00000004/art00015)	Article / URL
SPSS	IBM Corp, 2015; IBM SPSS Statistics for Windows, Version 23.0 (Version 23.0). Armonk, NY, United States	Software
R	R Core Team, 2019; R Foundation for Statistical Computing. Vienna, Austria. (Software retrieved from https://www.R-project.org/)	Software
SINGLIMS. EXE	Single case statistics:<br>Crawford, J. R., & Howell, D. C. (1998). Comparing an individual's test score against norms derived from small samples. *Clinical Neuropsychologist, 12*(4), 482-486. doi: 10.1076/clin.12.4.482.7241;<br>Crawford, J. R., & Garthwaite, P. H. (2002). Investigation of the single case in neuropsychology: confidence limits on the abnormality of test scores and test score differences. *Neuropsychologia, 40*(8), 1196-1208. doi:10.1016/s0028-3932(01)00224-x;<br>Crawford, J. R., Garthwaite, P. H., & Porter, S. (2010). Point and interval estimates of effect sizes for the case-controls design in neuropsychology: rationale, methods, implementations, and proposed reporting standards. *Cogn*	Articles/ HTML link/ Software

	Neuropsychol, *27*(3), 245-260. doi:10.1080/02643294.2010.513967; Software retrieved from https://homepages.abdn.ac.uk/j.crawford/pages/dept/abnolims.htm	
**Analysis Methods**		
Speech reception threshold	Current manuscript; Approach described by Fruend, I., Haenel, N. V., & Wichmann, F. A. (2011). Inference for psychometric functions in the presence of nonstationary behavior. *Journal of Vision*, *11*(6). doi: 10.1167/11.6.16	Text file (.doc)/ Article
fNIRS preprocessing steps	Current manuscript: Figure 3	Text file (.doc)/ Figure
ROI analysis	Current manuscript;	Text file (.doc)
